# Functional implication for myelin regeneration in recovery from ischaemic stroke

**DOI:** 10.1093/brain/awae080

**Published:** 2024-04-04

**Authors:** Stavros Vagionitis, Ragnhildur Thóra Káradóttir

**Affiliations:** Cambridge Stem Cell Institute and Department of Veterinary Medicine, University of Cambridge, Cambridge, CB2 0AW, UK; Cambridge Stem Cell Institute and Department of Veterinary Medicine, University of Cambridge, Cambridge, CB2 0AW, UK; Department of Physiology, BioMedical Center, Faculty of Medicine, University of Iceland, 101 Reykjavik, Iceland

## Abstract

This scientific commentary refers to ‘Prolonged myelin deficits contribute to neuron loss and functional impairments after ischaemic stroke’ by Cheng *et al*. (https://doi.org/10.1093/brain/awae029).

**This scientific commentary refers to ‘Prolonged myelin deficits contribute to neuron loss and functional impairments after ischaemic stroke’ by Cheng *et al*. (https://doi.org/10.1093/brain/awae029).**

Ischaemic stroke causes damage to both grey and white matter, leading to cellular death in the lesion area and loss of brain function. In recent years, our understanding of the role of glia in neurological diseases has dramatically increased, and the contribution of myelin to conditions beyond primary demyelinating diseases has started to emerge.^1^ Myelin, produced by oligodendrocytes, is a fatty layer around neurons that is essential for fast neuronal communication and neuronal health.^2^ The effect of ischaemia on glial function, and in particular on myelin and oligodendrocytes, has been extensively studied in animal models, with results revealing that vulnerability of specific oligodendrocytes and myelin to ischaemic insults can contribute to axonal damage.^3^

In contrast, the role of myelin and oligodendrocytes in recovery after stroke has received far less attention. Increased neuroplasticity, such as surviving neurons replacing lost connections, is important for improved functional outcomes after ischaemia.^4^ Mirroring neuroplasticity, there is increasing evidence that myelin itself also displays plasticity, and this plasticity has been implicated in learning.^5^ Myelin plasticity, like myelin regeneration, can involve the differentiation of oligodendrocyte precursor cells (OPCs) into new myelinating oligodendrocytes.^5^ However, the importance of myelin plasticity in the functional recovery following ischaemic stroke is underexplored.

In this issue of *Brain*, Cheng and co-workers^6^ set out to tackle this question by evaluating the extent of myelin damage, the functional implications of myelin loss and repair, and whether it is possible to promote myelin regeneration for improved functional outcomes. They use an impressive range of methods, combining studies in post-mortem tissue from individuals who have suffered stroke lesions in the basal ganglia with cellular characterisation in the transient middle cerebral artery occlusion (tMCAO) mouse model, where ischaemic lesions also occur in the basal ganglia. They confirm many previous observations regarding the effect of ischaemia on the cells within ischaemic lesions. They also interrogate functional and behavioural changes *in vivo*, using a new generation of transgenic fate mapping tools to provide novel mechanistic insights. However, where this study^6^ sets itself apart from others is that the results provide functional evidence for myelin regeneration preserving neuronal density and function, and improving behavioural outcomes after stroke (Fig. 1).

The findings are consistent with other studies of mouse models and post-mortem human brain, which show loss of myelin and reduced neuronal and oligodendrocyte cell densities within the ischaemic lesion. Post-mortem analysis in general is difficult to interpret as it does not reveal the temporal sequence of events nor allow for manipulations to test for causality. Myelin loss detected in post-mortem human brain samples alongside reduced neuronal cell density is often assumed to be a consequence of reduced neuronal health. To interrogate the role of myelin and the temporal sequence of events, Cheng and colleagues^6^ used novel transgenic lines that distinguish myelinating oligodendrocytes that existed pre-lesion from those that are newly differentiated from OPCs. They found that more than 60% of myelinating striatal oligodendrocytes disappeared after stroke, leaving some striatal axons demyelinated. They detected a small amount of myelin regeneration by electron microscopy (EM), but no difference in neuronal cell density. Although this type of EM analysis is difficult to perform accurately, in combination with genetic-fate mapping of OPCs entering the lesion, they were able to detect a small number of newly differentiated myelinating oligodendrocytes. This underscores the likelihood that myelin regeneration can occur in ischaemic lesions, but to a much lesser extent than is seen in models of effective myelin regeneration, such as those often used in multiple sclerosis research.^1,3,7^ This rapid proliferative response of OPCs alongside their failure to differentiate into myelinating oligodendrocytes is a common observation in ischaemic lesions, and studies have tried to determine ways of releasing this block of OPC differentiation.^3,7^ The severity of oligodendrocyte and neuronal loss and of OPC differentiation failure increases with age,^1,3^ and as observed in this study too,^6^ correlates with worsening of behavioural outcomes after ischaemic stroke in older animals compared to young.

To date it has been difficult to distinguish between the effects of neurons dying versus the consequences of myelin and oligodendrocyte loss for functional outcomes after stroke. Cheng and co-workers^6^ addressed this issue by using an OPC-specific Cre-Lox strategy to either prevent or promote OPC differentiation after tMCAO and then evaluating the cellular and functional effects. OPC differentiation failure induced by conditional knockout of the transcription factor Olig2—which defines the oligodendrocyte lineage and is necessary for oligodendrocyte differentiation—exacerbated neuronal loss and worsened outcomes in a battery of behavioural tasks (open field, beam walking, novel object recognition tasks, and Morris water maze). Conversely, promoting OPC differentiation by conditionally knocking out a negative regulator, the M1 muscarinic receptor (M1R),^8^ promoted neuronal survival and plasticity, and led to an increase in the number of myelinating oligodendrocytes, along with significantly improved outcomes in the beam walking, novel object recognition and Morris water maze tasks.

Remarkably these results indicate that OPC differentiation and myelin regeneration could play a critical role in functional recovery from tMCAO. We must consider that a conditional knockout approach can entail some ‘leakiness’ into neurons, and the authors do discuss and address this possibility. Moreover, although deleting Olig2 from OPCs prevents their differentiation, Olig2 is a major lineage-defining transcription factor responsible for the expression of a multitude of OPC-specific genes and OPC survival, and its deletion led to near elimination of OPCs.^6^ OPC differentiation-independent effects cannot therefore be ruled out in this study, particularly as OPCs have increasingly been implicated in roles relating to neuronal plasticity beyond their differentiation into myelinating oligodendrocytes.^9^ Nonetheless these results highlight a contribution of the oligodendrocyte lineage cells to recovery after tMCAO or stroke.

Promoting myelin regeneration or plasticity may thus represent a key therapeutic strategy for improving functional outcomes after stroke. Myelin regenerative therapies are already showing great promise,^10^ such as the antihistamine clemastine, which is also a muscarinic receptor antagonist and is known to promote OPC differentiation and remyelination.^8^ In the current study, treating mice with clemastine after tMCAO induction improved OPC differentiation, and promoted myelin regeneration and improvements in white matter structure.^6^ To determine the effect of enhanced myelin regeneration on neuronal survival and function, Cheng and co-workers^6^ switched to transgenic mice where neuronal cell survival could be genetically fate mapped along with *in vivo* multi-channel recordings in the striatum. Clemastine promoted neuronal survival and led to increased neuronal firing indicative of preserved function. These effects likely contributed to the significant improvement in behavioural measures of cognition observed in the mice. Clemastine treatment seemed to phenocopy the M1R conditional knock-out experiment, thus it is tempting to attribute clemastine’s effects to oligodendrocyte lineage cells and improved myelin regeneration. The enhanced neuronal survival, function and behavioural outcomes could also be due to direct effects on the neurons themselves. Regardless, these results highlight the possibility that myelin regenerative treatments may be effective for ischaemic stroke.

Overall, the results presented by Cheng and colleagues^6^ provide evidence that OPC differentiation and myelin regeneration promote neuronal health and functional recovery after stroke. Although the extent of the improvement is difficult to ascertain as comparison to control animals was missing, this study shows that myelin regenerative treatments currently in clinical trials for multiple sclerosis produce at least some preclinical functional improvement following stroke. This is a promising finding and highlights the importance of understanding the mechanisms of myelin regeneration and its therapeutic potential for stroke. Taking into consideration the fact that myelin plasticity is important for learning, memory and circuit function^5^ and that myelin provides metabolic support to neurons, it is certainly possible that myelin contributes to CNS disorders previously thought to be entirely neuronal. Myelin regenerative therapies may therefore represent a more widely applicable strategy for the treatment of a multitude of CNS conditions.

**Figure 1:**
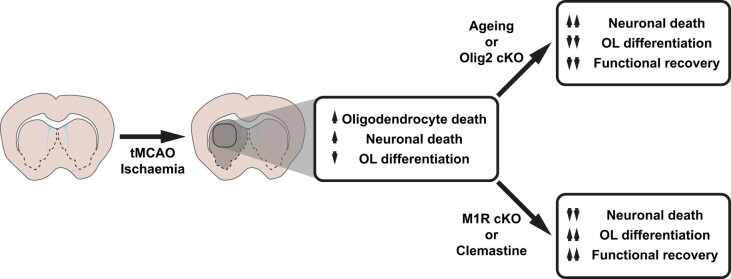
**Summary of the findings by Cheng and colleagues.^6^** Conditional knockout of the transcription factor Olig2—which is necessary for oligodendrocyte differentiation—exacerbated neuronal loss and worsened outcomes after tMCAO. Conversely, conditional knockout of the M1R—a negative regulator of oligodendrocyte differentiation—increased the number of myelinating oligodendrocytes and improved functional outcomes. Treatment with the promyelinating agent clemastine phenocopied the effects of M1R knockout. cKO = conditional knockout; M1R = M1 muscarinic receptor; OL = oligodendrocyte; tMCAO = transient middle cerebral artery occlusion.
